# The Striking Need for Age Diverse Pulse Oximeter Databases

**DOI:** 10.3389/fmed.2021.782422

**Published:** 2021-12-01

**Authors:** Mohamed Elgendi, Richard Ribon Fletcher, Harshit Tomar, John Allen, Rabab Ward, Carlo Menon

**Affiliations:** ^1^Biomedical and Mobile Health Technology Laboratory, Department of Health Sciences and Technology, ETH Zurich, Zurich, Switzerland; ^2^Menrva Research Group, School of Mechatronic Systems Engineering, Simon Fraser University, Vancouver, BC, Canada; ^3^Rady Faculty of Health Sciences, University of Manitoba, Winnipeg, MB, Canada; ^4^Department of Electrical and Computer Engineering, University of British Columbia, Vancouver, BC, Canada; ^5^Mechanical Engineering, Massachusetts Institute of Technology, Cambridge, MA, United States; ^6^Department of Psychiatry, University of Massachusetts Medical School, Worcester, MA, United States; ^7^Research Centre for Intelligent Healthcare, Coventry University, Coventry, United Kingdom

**Keywords:** digital health, pulse oximetry, intensive care, elderly population, anesthesia, photoplethysmography, PPG signal analysis, PPG waveform

## Introduction

The use of pulse oximetry data has grown significantly in recent years due to new applications of the technology and new wearable sensor platforms, as well as the widespread clinical demands of the ongoing coronavirus pandemic. The recent letter by Sjoding (1) (NEJM, Dec 2020) raising the effect of race (skin color) on pulse oximetry data has recently prompted the U.S. Food and Drug Administration (FDA) to exercise caution when using and interpreting pulse oximetry readings, with recommendation being given to following the trend in pulse oximeter readings rather than focusing on the absolute value of the readings alone (2). This finding is now being communicated to the nursing community as well (3).

The database referenced by Sjoding is one of many large pulse oximetry databases that are often used in clinical research to develop and decision support systems. In addition to the oxygen saturation values, there is now an increasing use of the morphological features of the pulse oximetry waveform which are being used, for example, to develop algorithms to predict blood pressure (4) as well as atherosclerosis (5) for use in patient monitoring and disease management. With the increasing use of these publicly available pulse oximetry databases, caution should be taken to prevent creating a bias in the resulting computer algorithms.

## Methods

Prompted by the Sjoding letter, we proceeded to perform a demographic analysis of the main publicly available pulse oximetry databases. In particular, we were most focused on age distribution across these data sets, since it is well-known that the pulse waveform morphology changes significantly as a function of age and atherosclerosis. The result of this analysis, using freely accessible databases (from 2013 through 2021) consisting of pulse oximeter signal (called photoplethysmogram or PPG) signals is presented in Table 1. We classified publicly available databases into two different age categories namely, children (<16 years) and adults (≥16 years).

**Table 1:** Details of different publicly available pulse oximeter databases.

**Database**	**No. of subjects**	**Age range (in years)**	**Purpose**	**Google scholar citations (as of 21^**st**^ of May 2021)**
BIDMC PPG and respiration dataset (6)	53	19–90+	Heart rate, respiration rate, and blood oxygen saturation level	101
Wrist PPG signals recorded during exercise (7)	8	22–32	Heart rate	39
MIMIC-III, a freely accessible critical care database (8)	53,423	16+	Heart rate, oxygen saturation, systolic, and diastolic blood pressure	2,641
Real-world PPG dataset (9)	35	NA	Heart rate	NA
PPG-BP database (10)	219	21–86	Blood pressure	45
PPG dalia (11)	15	21–55	Heart rate	43
WESAD (12)	15	24–35	NS	129
Synthetic dataset (13)	39	18–40	Respiratory rate	173
IEEEPPG dataset (14)	12	18–35	Heart rate	470
PULSE ID (15)	43	22–55	Biometric	14
CapnoBase (16)	42	<76	Respiratory rate	280

*NA, not available; NS, not specified*.

## Discussion

**Figure 1:**
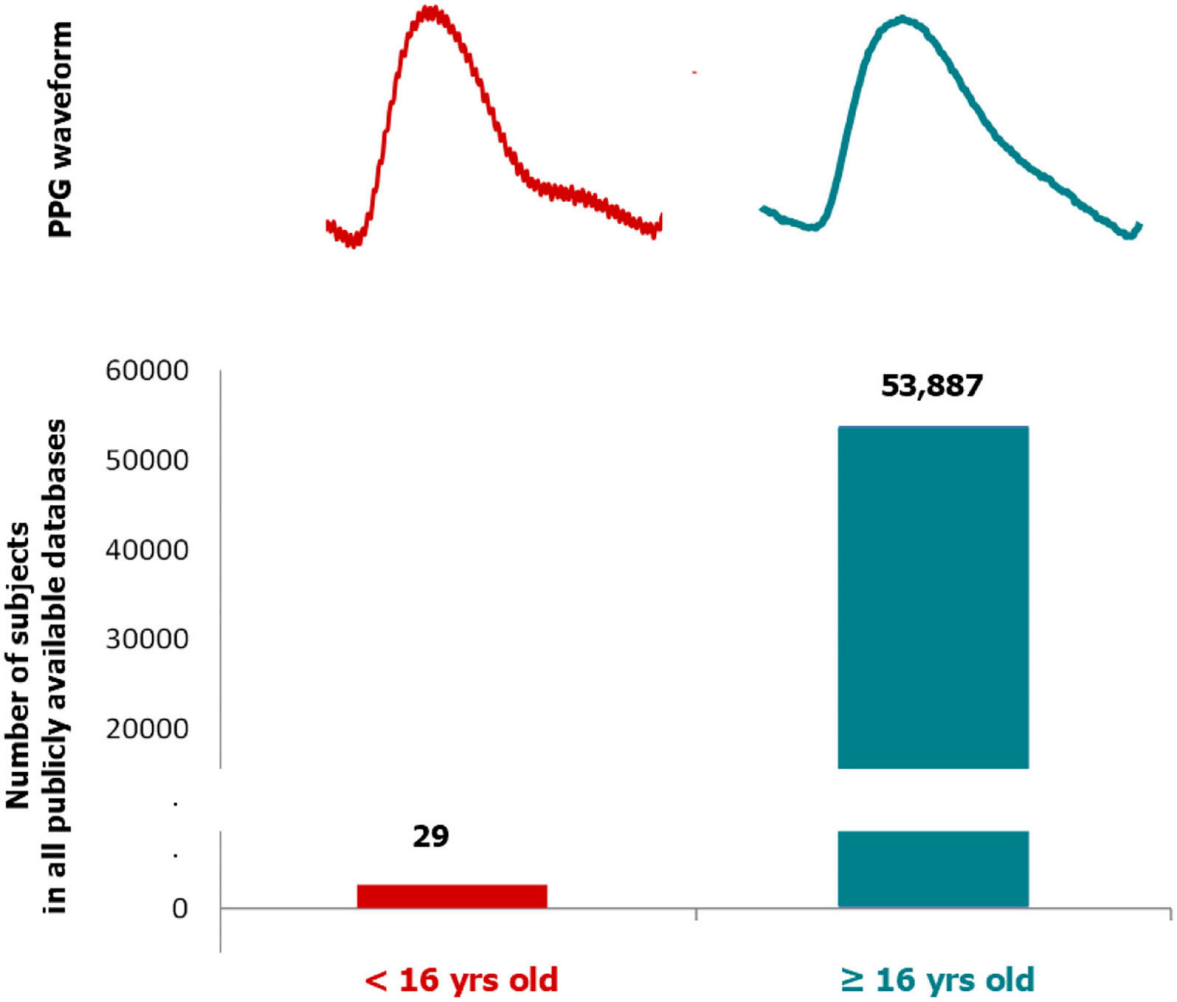
Number of subjects in publicly available pulse oximeter databases according to two age groups: children (<16 years) and adult (≥16 years). Two examples of photoplethysmogram waveforms collected via fingertip pulse oximetry probe (left: 15 years old and right: 50 years old) is shown for each age group. Note that the dicrotic notch is more salient in children compared to adults (i.e., the pulse becoming more triangulated with age).

As shown in Figure 1, there is a substantial difference in the number of subjects overall in all publicly available datasets between children and adults. This significant age bias could potentially impact algorithms developed to detect specific abnormalities. The morphology of the PPG waveform is typically different between children and adults, as shown in Figure 1. If a digital health solution is developed that combines a PPG sensor and an algorithm, then testing and evaluating over different age groups is essential to achieve reliability. Note that a significant difference (*p* < 0.05) between PPG waveform morphologies of different age groups (subjects younger than 30 years, 30–39 years, 40–49 years, and 50 years of age or older) was reported (17).

On examining all of the 12 databases as reported in Table 1, there is only one database (i.e., CapnoBase database) that has data recorded from the children age group, specifically 29 out of 42 subjects. Referring to all the above-mentioned reasons, it can be clearly stated that there is an age bias while recording data, which makes the evolution of devices such as pulse oximeters and systems for detecting vascular disease more biased toward the age category (16 years and above).

Most of the machine learning algorithms, developed to detect abnormalities, are published on publicly available databases. Even the FDA-approved PPG-based devices use publicly available databases for validation. If the publicly available databases are biased in terms of age, it is expected that all these algorithms will be developed for a specific age group. This point, to our knowledge, is not addressed by the FDA yet, and it is essential to raise awareness so researchers can add “in adults” in their titles, for example, or in the discussion.

We argue that since pulse oximetry measurements are particularly susceptible to age, caution should be taken when using these data to create computer algorithms for patient monitoring or diagnosis.

## Author Contributions

ME designed and led the study. ME, RF, HT, JA, RW, and CM conceived the study. All authors approved final manuscript.

## Funding

This work was supported by Canada Research Chair program and the Canadian Institutes of Health Research (CIHR) agency.

## Conflict of Interest

The authors declare that the research was conducted in the absence of any commercial or financial relationships that could be construed as a potential conflict of interest.

## Publisher's Note

All claims expressed in this article are solely those of the authors and do not necessarily represent those of their affiliated organizations, or those of the publisher, the editors and the reviewers. Any product that may be evaluated in this article, or claim that may be made by its manufacturer, is not guaranteed or endorsed by the publisher.
